# *LINC00909* up-regulates pluripotency factors and promotes cancer stemness and metastasis in pancreatic ductal adenocarcinoma by targeting *SMAD4*

**DOI:** 10.1186/s13062-024-00463-4

**Published:** 2024-03-19

**Authors:** Zhenchong Li, Zuyi Ma, Shujie Wang, Qian Yan, Hongkai Zhuang, Zixuan Zhou, Chunsheng Liu, Yubin Chen, Mingqian Han, Zelong Wu, Shanzhou Huang, Qi Zhou, Baohua Hou, Chuanzhao Zhang

**Affiliations:** 1 Department of General Surgery, Heyuan people’s Hospital, Heyuan, 517000 China; 2 Department of General Surgery, Guangdong Provincial People’s Hospital (Guangdong Academy of Medical Sciences), Southern Medical University, Guangzhou, 510080 China; 3 Junior Clinical Cooperation Unit Translational Gastrointestinal Oncology and Preclinical Models, German Cancer Research Center (DKFZ), 69120 Heidelberg, Germany; 4 Department of General Surgery, State Key Laboratory of Complex Severe and Rare Diseases, Peking Union Medical College Hospital, Chinese Academy of Medical Science and Peking Union Medical College, Beijing, 100730 China; 5 The Second School of Clinical Medicine, Southern Medical University, Guangzhou, Guangdong Province 510515 China; 6 South China University of Technology School of Medicine, Guangzhou, Guangdong Province 510006 China; 7 Department of Hepatobiliary Surgery, Sun Yat-sen Memorial Hospital, Sun Yat-sen University, Guangzhou, 510080 China; 8 Guangdong Provincial Key Laboratory of Malignant Tumor Epigenetics and Gene Regulation, Sun Yat-sen Memorial Hospital, Sun Yat-Sen University, Guangzhou, 510080 China; 9 Department of General Surgery, Hui Ya Hospital of The First Affiliated Hospital, Sun Yat-sen University, Huizhou, Guangdong 516081 China; 10 Department of Liver Surgery, The First Affiliated Hospital of Sun Yat-Sen University, Guangzhou, 510080 China

**Keywords:** *LINC00909*, Cancer stemness, Tumor metastasis, Pancreatic ductal adenocarcinoma, *SMAD4*, Pluripotency factors

## Abstract

**Background:**

Pancreatic cancer stem cells are crucial for tumorigenesis and cancer metastasis. Presently, long non-coding RNAs were found to be associated with Pancreatic Ductal Adenocarcinoma stemness characteristics but the underlying mechanism is largely known. Here, we aim to explore the function of *LINC00909* in regulating pancreatic cancer stemness and cancer metastasis.

**Methods:**

The expression level and clinical characteristics of *LINC00909* were verified in 80-paired normal pancreas and Pancreatic Ductal Adenocarcinoma tissues from Guangdong Provincial People’s Hospital cohort by in situ hybridization. RNA sequencing of PANC-1 cells with empty vector or vector encoding *LINC00909* was experimented for subsequent bioinformatics analysis. The effect of *LINC00909* in cancer stemness and metastasis was examined by in vitro and in vivo experiments. The interaction between *LINC00909* with *SMAD4* and the pluripotency factors were studied.

**Results:**

*LINC00909* was generally upregulated in pancreatic cancer tissues and was associated with inferior clinicopathologic features and outcome. Over-expression of *LINC00909* enhanced the expression of pluripotency factors and cancer stem cells phenotype, while knock-down of *LINC00909* decreased the expression of pluripotency factors and cancer stem cells phenotype. Moreover, *LINC00909* inversely regulated *SMAD4* expression, knock-down of SMAD4 rescued the effect of *LINC00909*-deletion inhibition on pluripotency factors and cancer stem cells phenotype. These indicated the effect of *LINC00909* on pluripotency factors and CSC phenotype was dependent on *SMAD4* and MAPK/JNK signaling pathway, another downstream pathway of *SMAD4* was also activated by *LINC00909.* Specifically, *LINC00909* was localized in the cytoplasm in pancreatic cancer cells and decreased the stability the *SMAD4* mRNA. Finally, we found over-expression of *LINC00909* not only accelerated tumor growth in subcutaneous mice models, but also facilitated tumorigenicity and spleen metastasis in orthotopic mice models.

**Conclusion:**

We demonstrate *LINC00909* inhibits *SMAD4* expression at the post-transcriptional level, which up-regulates the expression of pluripotency factors and activates the MAPK/JNK signaling pathway, leading to enrichment of cancer stem cells and cancer metastasis in pancreatic cancer.

**Supplementary Information:**

The online version contains supplementary material available at 10.1186/s13062-024-00463-4.

## Introduction

In terms of aggressiveness, pancreatic cancer (PC) is among the worst diseases of the digestive system; most patients present with vague symptoms until the disease reaches an advanced stage [1]. PC mortality has been rising in recent years, with a survival rate of 10% in the United States after 5 years [2]. High aggressiveness and early metastasis are crucial factors linked to inferior prognosis in pancreatic ductal adenocarcinoma (PDAC) patients [3, 4].

Tumor initiation and development are driven by a few colony of differentiated cells called cancer stem cells (CSCs). According to recent studies, pancreatic CSCs assume a crucial role during tumor recurrence, metastasis, and resistance to chemotherapy in PDAC [5]. There is evidence that the activation of epithelial-to-mesenchymal transition (EMT), a process associated with cancer cell metastasis, is linked to the properties of CSCs [6, 7]. Researchers have identified several pluripotent transcription factors, including *c-Myc*, *KLF4, OCT4*, *SOX2*, and *NANOG*, which are responsible for regulating stemness [8]. An abundance of CSCs and/or high expression of pluripotency factors in tumors may forebode worse prognosis in PDAC patients, indicating that repression of cancer stemness could provide a prospective means to improving the PDAC treatment. *SMAD4* is one of the most important driver genes in PDAC. This cancer suppressor gene is inactivated due to mutation or homozygous deletion in 60% of PDAC cases [9]. Inhibition of JUN N-terminal kinase (JNK) by *SMAD4* suggests that deletion of *SMAD4* would enhance JNK activity [10] and be required in the epithelial-to-mesenchymal transition response [11]. Importantly, *SMAD4* was found to affect the expression of pluripotency factors and regulate the CSC phenotype.

The term long non-coding RNA (LncRNA) refers to RNAs whose length exceeds 200 nt but lack the capability of coding proteins [12]. It has been demonstrated that lncRNAs mediated multi-faceted biological progression, such as immune response, cell differentiation, apoptosis, and cancer stemness [13]. For example, lncRNA-*HOTTIP* upregulated the expression of *HOXA9* as a result of activating the Wnt/β-catenin pathway, which is essential for maintaining the CSC phenotype in PDAC [14]. The level of long intergenic non-protein coding RNA 909 (*LINC00909*), a lncRNA with 15,407 bases length, is increased in glioma. Knockdown (KD) of *LINC00909* inhibited the proliferation of glioma cells, indicating that *LINC00909* drives tumor progression in glioma [15]. Nevertheless, the biological function of *LINC00909* in PDAC, in particular whether *LINC00909* contributes to regulating the CSC phenotype, is largely undiscovered.

As a result of this study, we identified that *LINC00909* was markedly upregulated in PDAC tissues. Moreover, *LINC00909* enhanced stemness and promoted metastasis in PDAC. Additionally, the increased expression of pluripotency factors induced by *LINC00909* was dependent on the downregulation of *SMAD4*. Our study suggests that *LINC00909* plays a vital role in controlling the CSC phenotype as well as cancer metastasis in PDAC.

## Materials and methods

### Identification of lncRNA

The Cancer Genome Atlas (TCGA) database (http//gdc.cancer.gov/) was used to obtain data on the expression of lncRNAs in 171 patients with PDAC.

### Patients and clinical samples

Eighty pairs of primary PDAC specimens were collected from patients undergoing surgical resection of PC at the Guangdong Provincial People’s Hospital (GDPH) between February 2008 and June 2021. After specimen collection, a temperature of 80 °C was used for the storage of all PDAC tissue samples. A summary of the clinicopathological characteristics of the patients is presented in Table S1. This study was authorized by the Ethics Committee of GDPH.

### Next-generation sequencing (NGS)

RNA was collected from PANC-1/Vector and PANC-1/*LINC00909*-overexpressing (PANC-1/*LINC00909*-OE) cells. Next-generation sequencing (NGS) was conducted as previously described [16].

### Functional enrichment analysis

According to the NGS results, we determined DEGs associated with *LINC00909* (adjusted *P* < 0.05 and Log_2_|fold change| >1.0). These DEGs were integrated into DAVID 6.8 (https://david-d.ncifcrf.gov/) and SangerBox (http://sangerbox.com/) for Gene Ontology (GO) analyses and Kyoto Encyclopedia of Genes and Genomes (KEGG) pathway analyses, respectively. Gene set enrichment analysis (GSEA) indicated the functional pathways and gene sets that exhibited obvious diversity between the high- and low-*LINC00909* expression groups.

## Materials

Gemcitabine (abs814679) was obtained from Univ (Shanghai, China). Antibodies against *CD133* (#64,326, 1/1,000), *c-Myc* (#5605, 1/1,000), *NANOG* (#4903, 1/2000), *SOX2* (#14,962, 1/1,000), *CD133* (#53,276, 1/50), and aldehyde dehydrogenase 1 family member A1 (*ALDH1A1*; #65,583, 1/50) were acquired from Cell Signaling Technology (Boston, MA, USA). Antibodies against *KLF4* (ab215036, 1/1,000), *OCT4* (ab181557, 1/1,000), *BCL-2* (ab32124, 1/1,000), p53-upregulated modulator of apoptosis (*PUMA*; ab33906, 1/2,500), *β-actin* (ab8226, 1/5,000), and *GAPDH* (ab8245, 1/2,000) were obtained from Abcam (Cambridge, UK). Antibodies against *SMAD4* (AF5247, 1/1,000), *p-JNK* (Thr183 + Thr185) (AF3318, 1/1,000),*XXXJNK* (AF6318), *p-JUN* (Ser73) (AF3095), *c-JUN* (AF6090, 1/1,000), *TWIST* (AF4009, 1/1000), *SNAIL* (AF6032, 1/1,000) were obtained from Affinity (Jiangsu Province, China). Actinomycin D (SBR00013) was obtained from Sigma–Aldrich (USA).

### Fluorescence in situ hybridization (FISH)

PDAC cells were collected, fixed with paraformaldehyde at 80–90% density, and pre-hybridized. Next, the Cy3-labeled *LINC00909* probe 5’-CTTTATCCACTCGTTGGAATGATTTTTTTGAGAC-3’ (Servicebio, Wuhan Province, China) was hybridized with hybridization buffer overnight at 37 °C. All pictures were taken by laser scanning confocal microscope (Olympus,Tokyo, Japan).

### Chromogenic in situ hybridization (CISH)

CISH were conducted using a PDAC and normal tissue micro-array. CISH was utilized for detecting *LINC00909* expression in tissue using a specific 5′- and 3′-digoxigenin-labeled probes. Using an organizational slicing digital scanner or imaging system, capture scan files or images of immunohistochemically stained tissue sections. With the help of an image analysis system, automatically extract measurements from the specified tissue areas. Classify positive staining into levels (i): Negative (0 score), Weakly positive (score 1, pale yellow), Moderately positive (score 2, brown-yellow), and strongly positive (score 3, brown-brown). Analyze weak, moderate, and strong positive areas, total tissue area, cumulative optical density (IOD) value of positive staining, and positive area within the measurement regions. Calculate these results to reflect the strength of positivity.

To assess positive staining intensity, we use the H-score method, tailored for specific slide characteristics. The H-score, short for Histochemistry score, converts positive staining quantity and intensity into numerical values, providing a semi-quantitative tissue staining assessment. The H-Score (H-SCORE = ∑(pi×i) = (percentage of weak intensity area ×1) + (percentage of moderate intensity area ×2) + (percentage of strong intensity area ×3)) employs pi for the positive signal pixel percentage and i for the positive staining level. H-score ranges from 0 to 300, with higher values indicating stronger overall positivity.

### Immunohistochemistry (IHC)

IHC were conducted using a PDAC and normal tissue micro-array. The tumor tissue was stained for *SMAD4*, *c-Myc*, *KLF4*, and *c-JUN*. The critical process was blocked 30 min in normal goat serum, then incubated with a primary antibody and a secondary antibody.

### PDAC cell lines and culture

The human PDAC cell lines (PANC-1, BxPC-3, CAPAN-2, AsPC-1, SW1990), and normal pancreas epithelium cell line (HPED-6) used in our study were obtained from Procell (Wuhan, China).

AsPC-1 and the other cells were respectively cultured in RPMI 1640 medium (Gibco, USA) and high-glucose DMEM (Gibco) with 10% fetal bovine serum (FBS) (Gibco) at 37 °C and 5% CO_2_.

### Lentivirus infection and siRNA KD

To establish stable OE cell lines, PANC-1 cells were infected with lentivirus [NR_024484.1] containing the EFS-h*LINC00909*-CMV-EGFP/T2A/Puro. After puromycin selection, we acquired stable OE cells. Following the instructions provided by manufacturer, we knocked down *LINC00909* and *SMAD4* by transfecting the siRNAs into PANC-1 or AsPC-1 cells. The siRNA sequences are shown in the **Table ****S1**.

### Proliferation assays

Cell Counting Kit-8 (CCK-8) and colony formation assays were utilized to measure cell proliferation. For the CCK-8 assays, a 96-well plate with 2*10^3^ PANC-1 and AsPC-1 cells per well was resuspended and cultured for 0, 24, 48, and 72 h. The CCK-8 reagent was added, and the cells were incubated for 2 h at 37 °C. Thereafter, the number of PDAC cells was determined by optical density at OD 450.

For the colony formation assays, PANC-1 or AsPC-1 cells were seeded into six-well plates (3,000 cells per well). These cells were cultivated in an incubator at 37 °C and 5% CO_2_ for 7 days. After rinsing with PBS, we stained the cells were with 0.1% crystal violet to observe the formation of colonies.

### Wound-healing and Transwell assays

When the cell density in the six-well plate reached 85%, we used a sterile pipette tip to make a scratch in the center of each well. Wound-healing results were observed at 0 and 24 h. The size of the wound was measured at least thrice.

For Transwell assays, 5 × 10^4^ PDAC cells were totally seeded in the upper chamber of Transwell plates with DMEM medium containing 10% FBS, while DMEM (800 µl) containing 20% FBS was injected into the lower chamber. After 24–48 h of incubation at 37 °C, the cells migrated from the upper chamber to the lower chamber. Subsequently, the upper chambers were taken out for washing thrice with PBS, then the cells were stained with 0.1% crystal violet. The amount of PDAC cells was calculated using microscope (Olympus,Tokyo, Japan).

### Sphere-formation assays

A total of 5 × 10^3^ PANC-1 or AsPC-1 cells were cultured in six-well ultra-low attachment plates (Corning, NY, USA) containing DMEM/F12 medium with B-27 supplement (1:50, Gibco), fibroblast growth factor (FGF; 20 ng/ml, Invitrogen, California, USA) and epidermal growth factor (EGF; 20 ng/ml, Invitrogen). Under a microscope (Olympus, Tokyo, Japan), tumor spheres with a diameter greater than 50 mm were counted and photographed after 7 days.

### RT-qPCR assays

The TRIzol reagent (Invitrogen, California, USA) was utilized to extract total RNA from PDAC tissues, human PDAC cell lines, and HPDE-6 cells. Total RNA (1–2 µg) was reverse-transcribed by High Capacity RNA-to-cDNA Kit (TakaRa, Tokyo, Japan). The primer sequences used for RT-qPCR were shown in the **Table S2**.

### Western blotting

For western blotting, these cells were collected and dissolved in RIPA buffer with 1% phenylmethylsulfonyl fluoride, 1% protease inhibitor, and 1% phosphatase inhibitor in an ice bath for 25 min to extract the protein. The BCA protein kit (#23,235; Thermo Fisher Scientific) was utilized to determine the protein concentration. On an 10% SDS-PAGE gel, the same amount of 30 µg protein was separated and transferred onto a polyvinylidene fuoride (PVDF) membrane. After blocking for 1 h in 5% skim milk, primary antibodies were incubated overnight at 4 °C with the PVDF membrane. Whereafter, the membranes were rinsed with TBST buffer five times (5 min per wash) and incubated with horseradish peroxidase-labeled goat anti-rabbit IgG secondary antibody. Finally, the enhanced chemiluminescence reagent was added onto the membranes to visualize the proteins using a Tanon-5200 Chemiluminescent Imaging System (Tanon, China).

## Results

### *LINC00909* was highly expressed in human PDAC and associated with worse clinicopathologic features and outcome

**Fig. 1:**
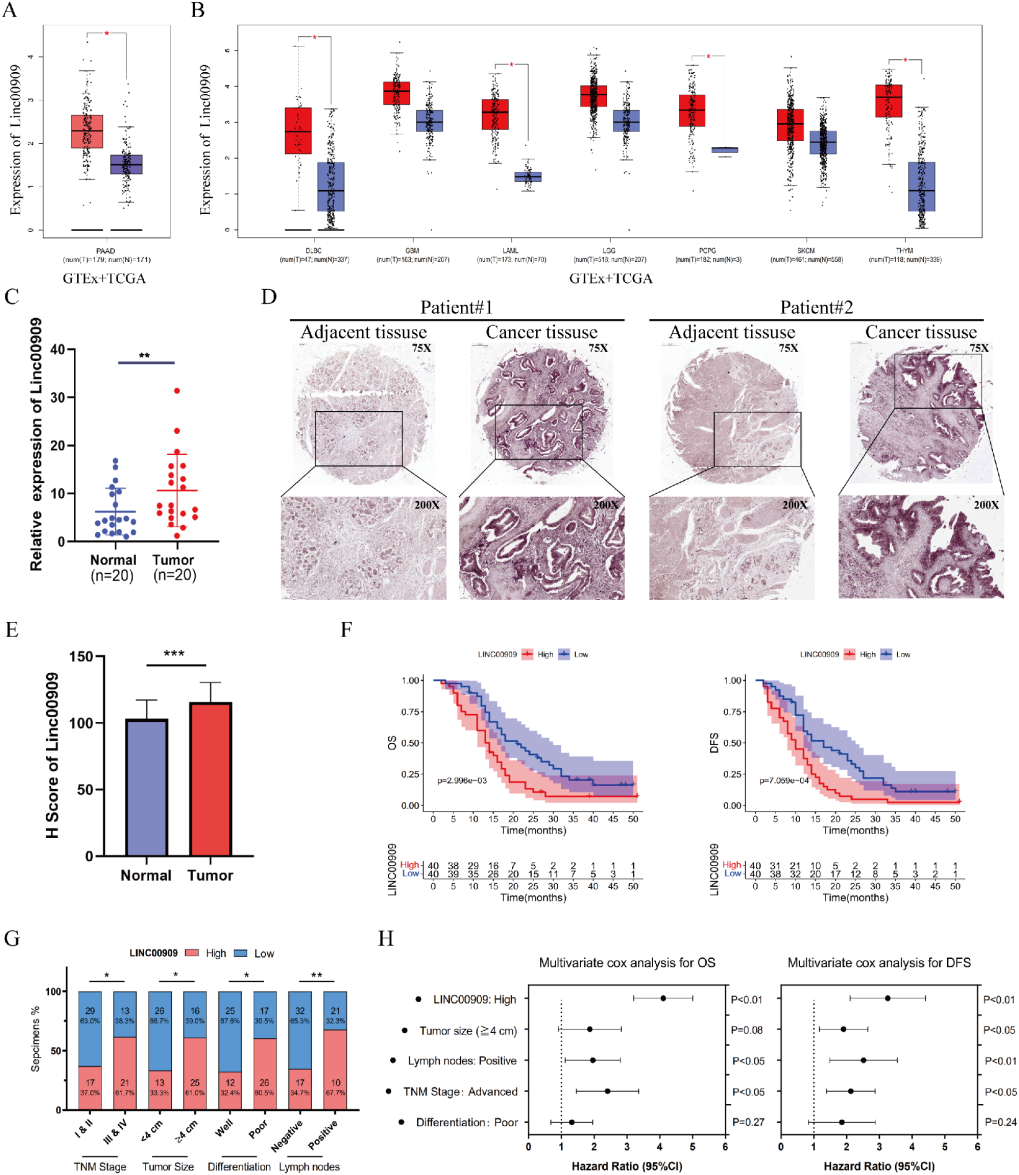
*LINC00909* is highly expressed in human pancreatic ductal adenocarcinoma (PDAC) and associated with worse clinicopathological features and outcome. (**A**) Upregulation of *LINC00909* in PDAC was verified using TCGA and GTEx data. (**B**) Analysis of data from the GEPIA database demonstrated that *LINC00909* expression was overexpressed in lymphoid neoplasm diffuse large glioblastoma multiforme (GBM), B-cell lymphoma (DLBC), brain lower grade glioma (LGG), acute myeloid leukemia (LAML), pheochromocytoma and paraganglioma (PCPG), thymoma (THYM) and skin cutaneous melanoma (SKCM). (**C**) RT-qPCR revealed high expression of *LINC00909* in human PDAC tissues compared with normal pancreas tissues (*n* = 20). (**D**) Representative images of *LINC00909* CISH analysis in the normal pancreas tissues (*n* = 80) and PDAC tissues (*n* = 80). (**E**) The H-Score of CISH analysis confirmed that the expression of *LINC00909* was upregulated in PDAC tissues. (**F**) Kaplan–Meier analyses representing the overall survival (OS) and disease-free survival (DFS) of patients with PDAC. These patients with high expression of *LINC00909* in the GDPH cohort were associated with poor prognosis. (**G**) *LINC00909* expression in PDAC was strongly correlated with advanced TNM stage, larger tumor size, poorer differentiation, and more lymph node metastasis. (**H**) Multivariate Cox regression analyses of 80 patients with PDAC from the GDPH cohort showed that high levels of *LINC00909* served as an independent risk factor for OS and DFS. All *P < 0.05; **P < 0.01; ***P < 0.001; ****P < 0.0001. *P*-values were caculated by the non-parametric Mann–Whitney *U*-test in (**A**) and (**B**). *P*-values were calculated by two-tailed *t*-tests in (**C**) and (**E**). *P*-values were assessed by χ^2^ tests or Fisher’s exact tests in (**G**). *P*-values and the hazard ratios (HR) by the log-rank (Mantel–Cox) test are calculated in (**H**)

We sought to assess the role of *LINC00909* in cancer progression. For this purpose, we used the GEPIA database (http://gepia.cancer-pku.cn/) to investigate whether *LINC00909* was obviously expressed in PDAC (Fig. 1A). We found that *LINC00909* was overexpressed in most types of cancer (Fig. 1B). To further verify the findings from the GEPIA database, the expression of *LINC00909* in 20 pairs of fresh PDAC tissues and adjacent normal tissues was subsequently examined by RT-qPCR analysis. Comparing with adjacent normal tissues, the results exhibited higher *LINC00909* mRNA expression in PDAC tissues (Fig. 1C). Moreover, CISH analysis and the H-score of CISH revealed higher expression of *LINC00909* in the glandular epithelium tissues of PDAC than in the adjacent normal tissues (Fig. 1D, E). CISH staining of these 80 pairs of PC and adjacent normal tissues was performed to evaluate the clinical role of *LINC00909*. Importantly, Kaplan–Meier analysis for the GDPH cohorts demonstrated that PDAC patients with high *LINC00909* expression were associated with poor overall survival (*P* = 2.996e-03) and disease-free survival (*P* = 7.059e-04) (Fig. 1F). Furthermore, patients with high *LINC00909* expression in other types of tumors (e.g., liver hepatocellular carcinoma, brain lower grade glioma, adrenocortical carcinoma, and bladder urothelial carcinoma) also had poor prognosis (**Fig. ****S1**).

Additionally, the correlation between *LINC00909* expression and the clinicopathological features of patients with PDAC indicated that *LINC00909* OE was correlated with advanced TNM stage (*P* < 0.05), larger tumor size (*P* < 0.05), poorer differentiation (*P* < 0.05), and more lymph node metastasis (*P* < 0.01) (**Table S3 and** Fig. 1G). Thereafter, the multivariate Cox regression analyses revealed that *LINC00909* served as a risk factor for poor prognosis in 80 GDPH patients with PDAC (**Tables S4, S5 and** Fig. 1H).

### *LINC00909* promoted PDAC cell proliferation and migration in vitro

**Fig. 2:**
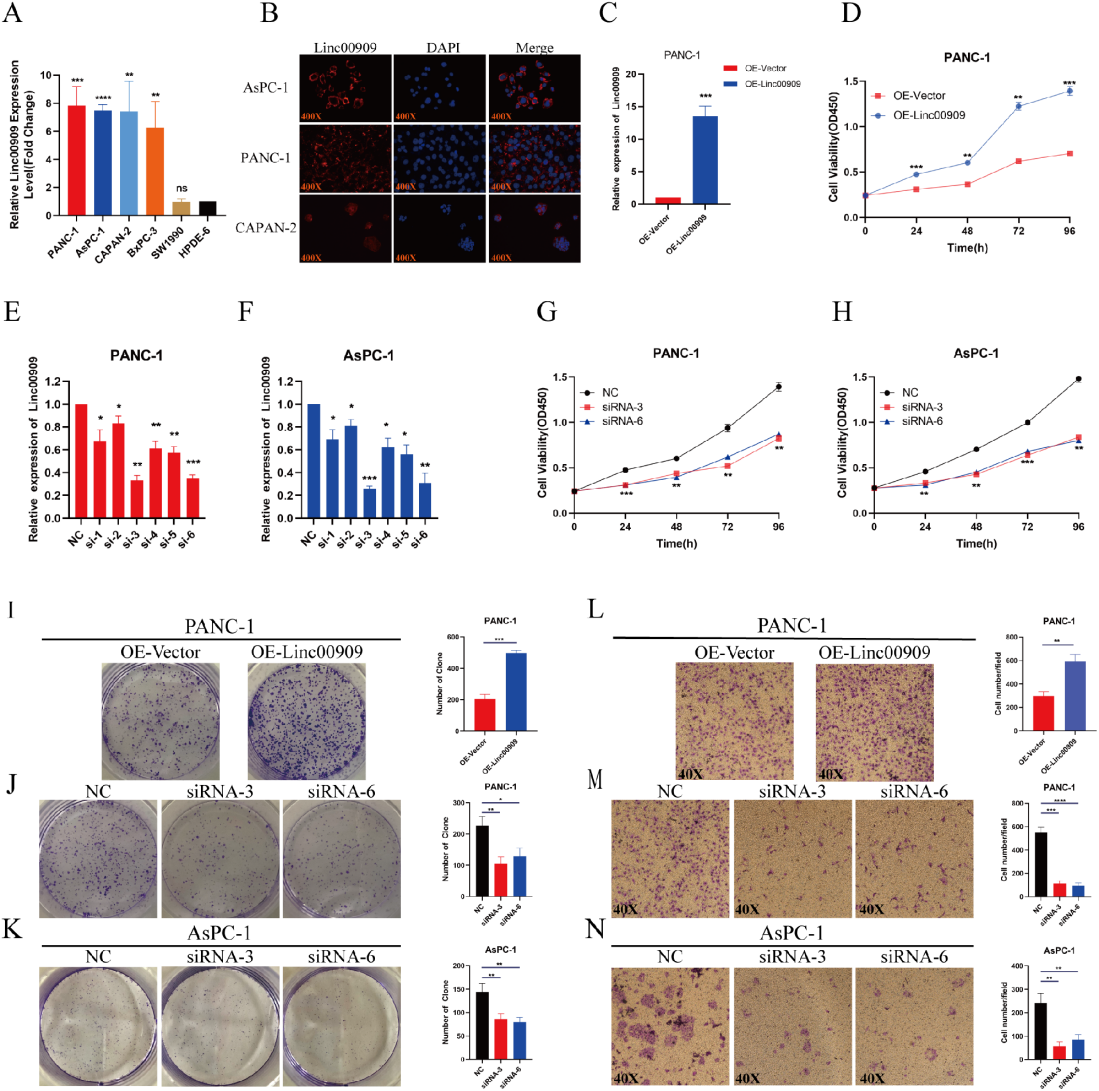
*LINC00909* promotes the proliferation and migration of pancreatic ductal adenocarcinoma (PDAC) cells. (**A**) RT-qPCR showed that *LINC00909* was highly expressed in PANC-1 and AsPC-1 cells among PDAC cell lines. (**B**) Identification of the cytoplasmic location of *LINC00909* in AsPC-1, PANC-1, and CAPAN-2 cells using FISH. (**C**) The efficiency of *LINC00909* overexpression in PANC-1 cells was assessed using RT-qPCR. (**D**) The effects of *LINC00909* overexpression on cell proliferation and viability were verified using CCK-8 assays. (**E**, **F**) The efficiency of *LINC00909* knockdown in PANC-1 and AsPC-1 cells was detected by RT-qPCR. (**G**, **H**) The effects of *LINC00909* knockdown on cell proliferation and viability were verified using CCK-8 assays. (I–K) The effect of *LINC00909* overexpression and knockdown on colony formation was investigated in PDAC cell lines. (L–N) Representative images of Transwell assays after *LINC00909* overexpression and knockdown in PDAC cell lines. All *P < 0.05; **P < 0.01; ***P < 0.001; ****P < 0.0001. *P*-values were assessed using ANOVA and two-tailed *t*-tests, followed by Dunnett’s tests for multiple comparisons in (**A**) and (**C**–**N**). All data represent the means ± SD from 3 independent assays

We performed RT-qPCR to monitor the expression of *LINC00909* in each cell line. The results showed that *LINC00909* had higher expression in PDAC cell lines (particularly PANC-1 and AsPC-1) compared with HPDE-6 cells (a normal pancreatic cell line) (Fig. 2A). FISH analysis was conducted to investigate the localization of *LINC00909* in PDAC cells. *LINC00909* RNA was principally located in the cytoplasm of PDAC cell lines (e.g., AsPC-1, PANC-1, and CAPAN-2) (Fig. 2B). Subsequently, a stable *LINC00909*-OE cell line (PANC-1/*LINC00909*-OE) was constructed to further analyze the gain-of-function of *LINC00909* (Fig. 2C). In contrast, six siRNAs of *LINC00909* were transiently transfected into PANC-1 and AsPC-1 cells to obtain *LINC00909-*KD cell lines (PANC-1/*LINC00909*-KD and AsPC-1/*LINC00909*-KD) for deletion-of-function analysis by gene KD (Fig. 2E, F). Subsequently, proliferation assays were performed using these three cell lines to evaluate the proliferative capability of these cells. As shown in Fig. 2D and I, upregulation of *LINC00909* significantly increased the proliferation of PANC-1 cells. On the contrary, downregulation of *LINC00909* prohibited the proliferative capacity of PANC-1/*LINC00909*-KD and AsPC-1/*LINC00909*-KD cell lines (Fig. 2G, H, J, K). We also performed Transwell and wound-healing assays to assess the functions of *LINC00909* on cell migration. These migration assays indicated that OE of *LINC00909* augmented the migration of PANC-1/*LINC00909*-OE cells (Fig. 2L and S2A), whereas silencing of *LINC00909* weakened the migratory ability of tumor cells in KD cell lines in vitro (Fig. 2M, N and S2B, C).

### *LINC00909* was associated with the MAPK/JNK pathway and pluripotency factors in PDAC

**Fig. 3:**
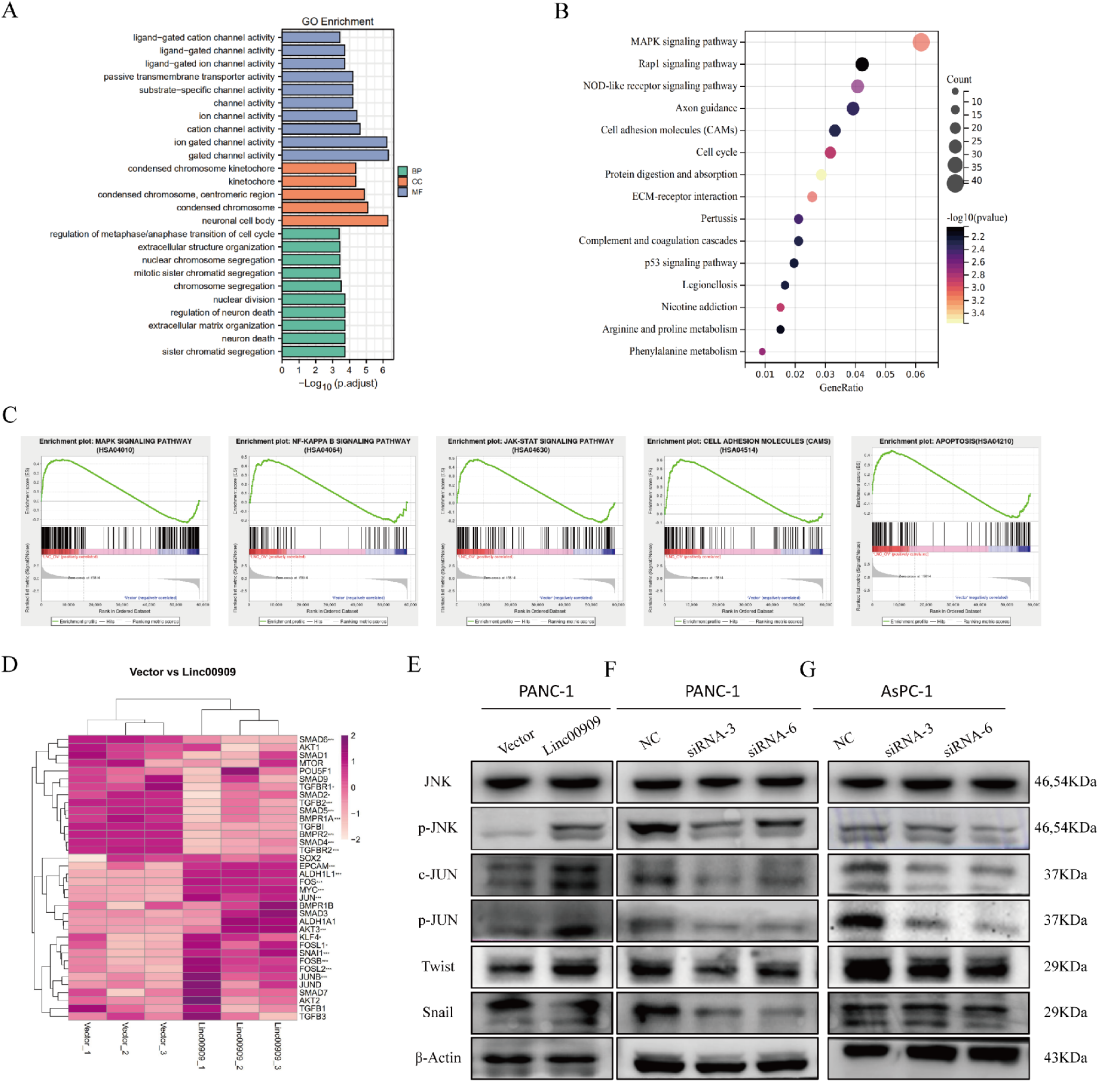
*LINC00909* is associated with the MAPK/JNK pathway and pluripotency factors in pancreatic ductal adenocarcinoma (PDAC). (**A**) Gene Ontology (GO) enrichment analysis of *LINC00909* based on the NGS database. (**B**) Top 15-enriched Kyoto Encyclopedia of Genes and Genomes (KEGG) pathways of *LINC00909* based on the NGS database. (**C**) Gene set enrichment analysis (GSEA) of the NGS database. (**D**) The expression of factors associated with stemness and metastasis was analyzed by NGS using PANC-1/Vector and PANC-1/*LINC00909*-OE cells. These factors are shown in the heatmap. (**E**–**G**) Western blotting analysis was conducted to detect the protein expression of *JNK* phospho-*JNK*, *c-JUN*, phospho-*JUN*, *TWIST*, and *SNAIL* in *LINC00909*-overexpressing cells (**E**) and *LINC00909*-knockdown cells (**F**, **G**). All *P < 0.05; **P < 0.01; ***P < 0.001; ****P < 0.0001. *P*-values were assessed using ANOVA and two-tailed *t*-tests, followed by Dunnett’s tests for multiple comparisons in (**D**)

To further explore the function of *LINC00909*, 2265 DEGs (Log_2_|fold change| >1.0 and adjusted *P-*value < 0.05) obtained from the NGS database of PANC-1/Vector and PANC-1/*LINC00909*-OE cells were inputted into DAVID 6.8 and SangerBox for functional enrichment analyses, respectively. The DEGs data was stored in **Table S6**. The GO analysis showed that *LINC00909* and the DEGs were principally enriched in “extracellular matrix organization”, “nuclear division”, “extracellular structure organization”, “regulation of metaphase/anaphase transition of cell cycle”, and “kinetochore” (Fig. 3A). The KEGG pathway enrichment analysis indicated that *LINC00909* was likely participate in “MAPK signaling pathway”, “cell adhesion molecules”, “cell cycle”, and “*P53* signaling pathway” (Fig. 3B). Meanwhile, GSEA was also carried out to identify the DEGs and pathways distinguishing between the -high and -low expression of *LINC00909*. No surprise, biological processes were significantly enriched in “MAPK signaling pathway”, “NF-κB signaling pathway”, “JAK-STAT signaling pathway”, “cell adhesion molecules”, and “apoptosis” (Fig. 3C).

Hence, the mRNA expression of essential molecules associated with the MAPK signaling pathway and other potential pathways were analyzed according to the NGS. We found that the expression of *JUN* and *FOS* in the MAPK pathway was upregulated. Interestingly, pluripotency factors *c-Myc*, *KLF4*, and stem cell markers (*EPCAM* and *ALDH1*) were also upregulated (Fig. 3D). Next, we utilized western blotting to verify the protein expression of phospho-*JNK*, as well as its downstream and metastasis-related molecules to explore the effect of *LINC00909* on the MAPK/JNK pathway. And the results disclosed that the protein expression of phospho-*JNK*, *c-JUN*, phospho-*JUN*, and tumor metastasis-related molecules (e.g., *TWIST* and *SNAIL*) was obviously enhanced in PANC-1/*LINC00909*-OE cells (Fig. 3E), whereas it was decreased in the PANC-1/*LINC00909*-KD and AsPC-1/*LINC00909*-KD cell lines (Fig. 3F, G). These results demonstrated that *LINC00909* was associated with the MAPK/JNK pathway and pluripotency factors in PDAC.

### *LINC00909* upregulated the expression of pluripotency factors and promoted the pancreatic CSC phenotype

**Fig. 4:**
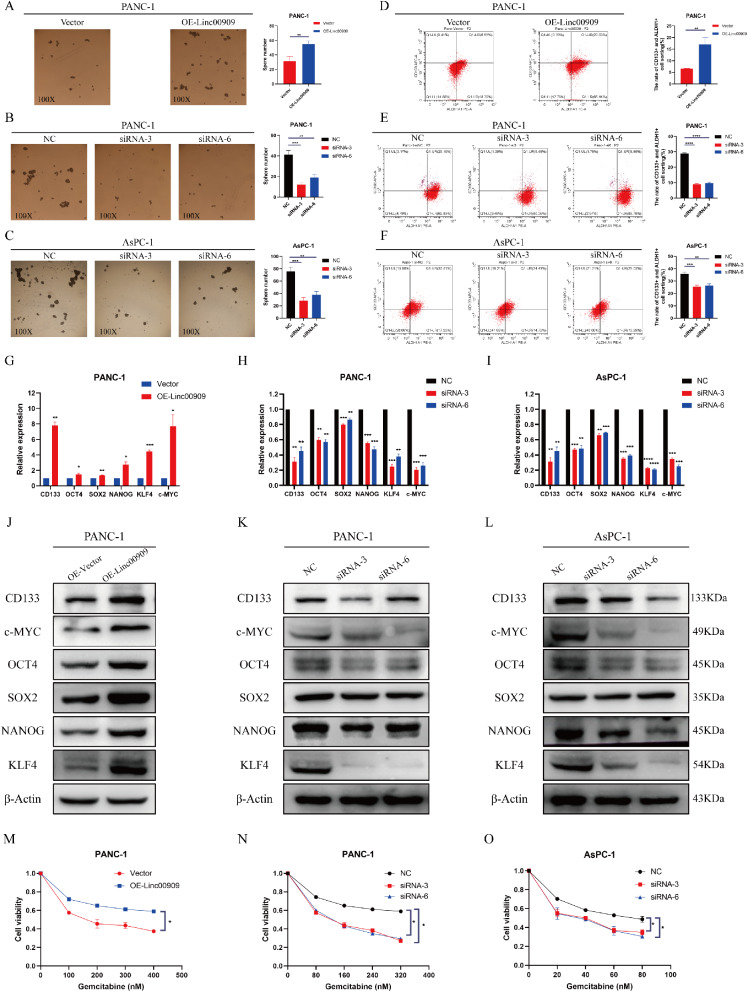
*LINC00909* promotes the pancreatic CSC phenotype and upregulates the expression of pluripotency factors. (**A**) Sphere-formation assay in PANC-1 cells transfected with empty vector (EV) or vector encoding *LINC00909*. (**B**, **C**) Sphere-formation assay in PANC-1 and AsPC-1 cells transfected with siNC, si*LINC00909*-3, or si*LINC00909*-6. (**D**, **F**) *CD133* + and *ALDH1* + expression in PDAC cells was measured by flow cytometry. (**G**–**I**) RT-qPCR analysis was conducted to detect the mRNA levels of *CD133*, *OCT4*, *SOX2*, *NANOG*, *KLF4*, and *c-Myc* in *LINC00909*-overexpressing cells (**G**) and *LINC00909*-knockdown cells (**H**, **I**). (**J**–**L**) Western blotting analysis was conducted to detect the protein expression of *CD133*, *OCT4*, *SOX2*, *NANOG*, *KLF4*, and *c-Myc* in PDAC cells. (**M**) CCK-8 assays were conducted to detect the cell viability of PANC-1 cells transfected with EV or vector encoding *LINC00909* under treatment with different concentrations of gemcitabine. (**N**, **O**) CCK-8 assays were conducted to detect the cell viability of PANC-1 or AsPC-1 cells transfected with siNC, si*LINC00909*-3, or si*LINC00909*-6 under treatment with different concentrations of gemcitabine. All *P < 0.05; **P < 0.01; ***P < 0.001; ****P < 0.0001. *P*-values were assessed using ANOVA and two-tailed *t*-tests, followed by Dunnett’s tests for multiple comparisons in (**A**–**I**) and (**M**–**O**).

We performed sphere-formation assays in three PDAC cell lines to investigate whether *LINC00909* regulates the pancreas CSC phenotype. From the results, *LINC00909*-OE significantly augmented the amount of PANC-1 cell spheres (Fig. 4A), whereas *LINC00909*-KD seriously impaired the sphere-formation capability of PANC-1 and AsPC-1 cells (Fig. 4B, C). We also measured the stem cell populations of *CD133* and *ALDH1* double-positive cells using flow cytometry. OE of *LINC00909* markedly augmented the double-positive rate of *CD133*^*+*^ and *ALDH1*^*+*^ (Fig. 4D), whereas KD of *LINC00909* reduced the expression of these markers (Fig. 4E, F). Next, we explored the effect of *LINC00909* on the expression of pluripotency factors. The GEPIA database analysis suggested that *LINC00909* was positively correlated with several stemness factors (**Fig. S4A**). According to RT-qPCR and western blotting results, the mRNA and protein levels of these stemness factors, particularly *c-Myc*, *KLF4*, and *CD133*, were drastically enhanced in PANC-1/*LINC00909*-OE (Fig. 4G, J). In contrast, they were noticeably reduced after the deletion of *LINC00909* in PANC-1 and AsPC-1 cells (Fig. 4H, I, K, L). In summary, these data manifested that *LINC00909* affects the expression of pluripotency factors and is necessary for maintaining CSC properties in PDAC.

CSCs contribute to the development of chemo-resistance in cancer. We next investigated whether *LINC00909* affects the chemo-sensitivity of PDAC cells. Gemcitabine, a pyrimidine anti-tumor agent commonly used in patients with advanced PC, was employed in the in vitro experiments. The results showed that *LINC00909*-OE lessened the sensitivity of PANC-1 cells to gemcitabine, as evidenced by the elevated cell viability following treatment with gemcitabine (Fig. 4M). Conversely, suppression of *LINC00909* sensitized PANC-1 and AsPC-1 cells to gemcitabine, as reflected by the declining cell viability (Fig. 4N, O). These data indicated that OE of *LINC00909* leads to chemo-resistance in PDAC.

### *LINC00909* enhanced the stemness of PDAC by inhibiting SMAD4 expression

We next investigated the underlying mechanism through which *LINC00909* regulates the expression of pluripotency factors. *LINC00909* is primarily distributed in the cytoplasm, while pluripotency factors (e.g., *KLF4* and *c-Myc*) are transcription factors localized in the nucleus. Therefore, we hypothesized that *LINC00909* may not physically interact and directly regulate these pluripotency factors.

**Fig. 5:**
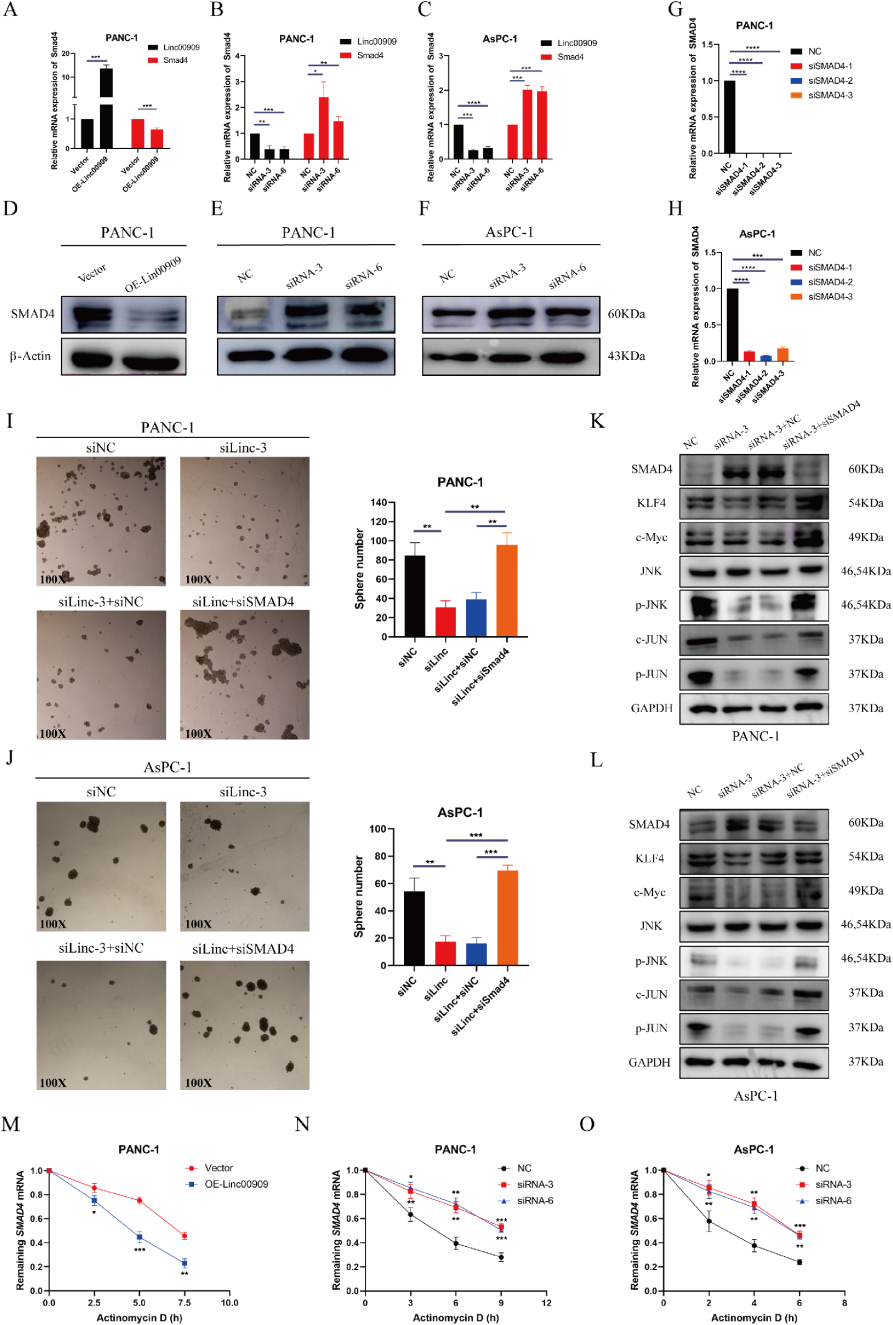
*LINC00909* enhances the stemness of pancreatic ductal adenocarcinoma (PDAC) by inhibiting *SMAD4* expression. (**A**–**C**) RT-qPCR analysis was performed to detect the mRNA levels of *SMAD4* in *LINC00909*-overexpressing cells (**A**) and *LINC00909*-knockdown cells (**B**, **C**). (**D**–**F**) Western blotting analysis was conducted to examine the protein expression of *SMAD4* in PDAC cells. (**G**, **H**) The efficiency of *SMAD4* knockdown in PANC-1 (**G**) and AsPC-1 cells (**H**) was detected by RT-qPCR. (**I**, **J**) Representative images of sphere-formation assays in PANC-1/KD and AsPC-1/KD cells transfected with si*SMAD4*-2. (**K**, **L**) The protein levels of **SMAD4**, *KLF4*, *c-Myc*, *JNK*, *p-JNK*, *JUN* and *p-JUN* in PANC-1/*LINC00909*-KD (**K**) and AsPC-1/*LINC00909*-KD cells (**L**) transfected with si*SMAD4*-2 were analyzed by western blotting. (**M**) PANC-1 cells transfected with EV or vector encoding *LINC00909* were treated with actinomycin D (1 mg/ml) at the indicated time point. (**N**, **O**) PANC-1 or AsPC-1 cells transfected with siNC, si*LINC00909*-3, or si*LINC00909*-6 were treated with actinomycin D (1 mg/ml) at the indicated time point. Total RNA was extracted and analyzed by RT-qPCR to examine the relative levels of *SMAD4* mRNA (M–O). All *P < 0.05; **P < 0.01; ***P < 0.001; ****P < 0.0001. *P*-values were assessed using ANOVA and two-tailed *t*-tests, followed by Dunnett’s tests for multiple comparisons in (**A**–**O**).

**Fig. 6:**
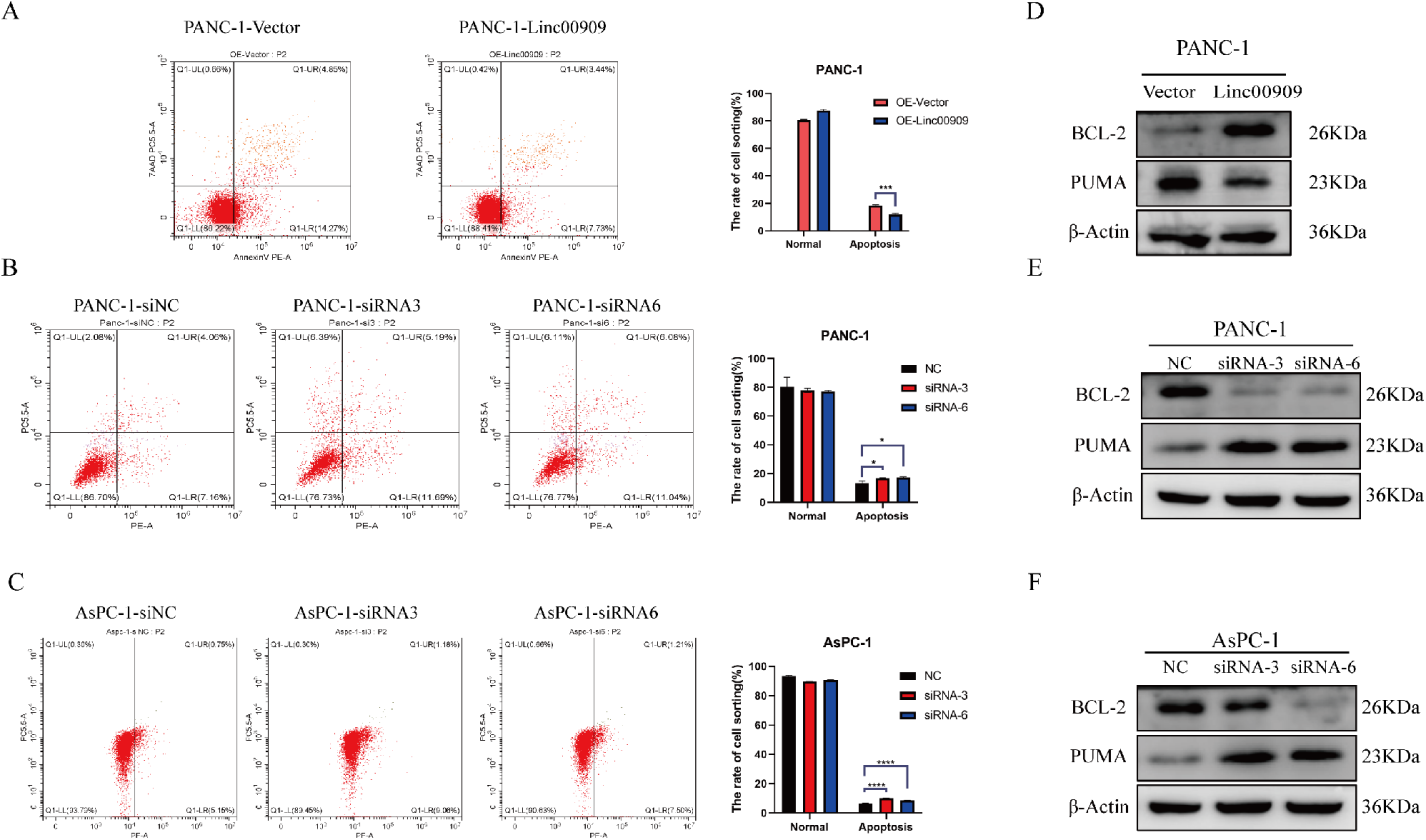
Overexpression of *LINC00909* promotes apoptosis in PDAC cells. (**A**) Flow cytometry was conducted to detect apoptosis after overexpression of *LINC00909* in PANC-1 cells. (**B**, **C**) Flow cytometry was utilized to detect apoptosis after knockdown of *LINC00909* in PANC-1 and BxPC-3 cells. (**D**) Western blotting was performed to examine the expression of *BCL-2* and *PUMA* after overexpression of *LINC00909* in PANC-1 cells. (**E**, **F**) Western blotting was performed to examine the expression of *BCL-2* and *PUMA* after knockdown of *LINC00909* in PANC-1 and BxPC-3 cells. *P*-values were assessed using two-tailed *t*-tests in (A–C). (***P < 0.001; ****P < 0.0001). *BCL-*2: B-cell lymphoma-2; *LINC00909*: Long intergenic non-protein coding RNA 00909; PDAC: Pancreatic ductal adenocarcinoma

The results of RNA sequencing showed that *SMAD4* was downregulated in PANC-1/*LINC00909*-OE cells, and *SMAD4* regulates the expression of pluripotency factors. Therefore, we investigated the levels of *SMAD4* to further identify correlations with *LINC00909.* RT-qPCR and western blotting analysis confirmed that both the mRNA and protein levels of *SMAD4* were declined in *LINC00909-*OE cells (Fig. 5A, D), while *SMAD4* was upregulated after deletion of *LINC00909* (Figs. 5B and C and 6E and F). To further investigate whether *SMAD4* deficiency contributes to the *LINC00909*-mediated enhancement of stemness in PDAC cells, we knocked down *SMAD4* in PANC-1/*LINC00909*-KD and AsPC-1/*LINC00909*-KD cells and performed sphere-formation assays. The KD efficiency of *SMAD4* in these two cell types was confirmed by RT-qPCR, and si*SMAD4*-2 was selected for subsequent experiments (Fig. 5G, H). As shown in sphere-formation assays, the downregulation of *SMAD4* transparently enhanced the reduction of spherical formation mediated by *LINC00909* deficiency in PANC-1 and AsPC-1 cells (Fig. 5I, J). Intriguingly, the western blotting analysis revealed that *SMAD4* deficiency markedly augmented the decrease in *KLF4*, *c-Myc*, *p-JNK* and *p-JUN* expression mediated by *LINC00909-*KD (Fig. 5K, L). These consequences confirmed that the regulatory effect of *LINC00909* on pluripotency factors, cancer stemness and the activation of the MAPK/JNK pathway were dependent on the inhibition of *SMAD4*.

LncRNAs localized in the cytoplasm act as a momentous role in post-transcriptional gene regulation (e.g., mRNA stability and protein modification). We hypothesized that *LINC00909* affects *SMAD4* mRNA stability since both the mRNA and protein levels were changed in *LINC00909-*KD or -OE cells. We treated cells with actinomycin D to inhibit general gene transcription and performed RT-qPCR at the indicated time point to explore the turnover of *SMAD4* mRNA. The results showed that OE of *LINC00909* accelerated the turnover of *SMAD4* mRNA (Fig. 5M), while KD of *LINC00909* exerted the opposite effect (Fig. 5N, O). These results imply that *LINC00909* inhibits SMAD4 expression by decreasing *SMAD4* mRNA stability.

### *LINC00909* inhibited apoptosis in PC cells.

According to the GSEA analysis, the biological process “apoptosis” was significantly enriched (Fig. 3C); four genes associated with apoptosis were selected to analyze their correlation with *LINC00909* in TCGA dataset using the GEPIA database. The analysis predicted that the levels of *LINC00909* were positively correlated with *BCL2* (correlation [Cor] = 0.41, *P* = 7.8e-09), and negatively correlated with BCL2 binding component 3 (*BBC3*; Cor = − 0.33, *P* = 7.8e-06), BCL2 antagonist/killer 1 (*BAK1*; Cor = 0.3, *P* = 3.4e-05), and BH3 interacting domain death agonist (*BID*; Cor = − 0.26, *P* = 0.00055) (**Fig. S3B)**. Subsequently, the consequences of flow cytometry confirmed that *LINC00909*-OE distinctly decreased the amount of apoptotic PANC-1 cells (Fig. 6A); however, *LINC00909*-KD obviously increased the quantity of apoptotic PANC-1 and AsPC-1 cells (Fig. 6B, C). Furthermore, we conducted a western blotting analysis to verify that *LINC00909*-OE upregulated the expression of BCL-2 and suppressed the levels of PUMA in PANC-1 cells (Fig. 6D). Inversely, downregulation of *LINC00909* increased the levels of PUMA and inhibited BCL-2 expression (Fig. 6E, F). These data showed that *LINC00909* repressed apoptosis in PDAC.

### *LINC00909* accelerated tumor growth and metastasis in vivo

**Fig. 7:**
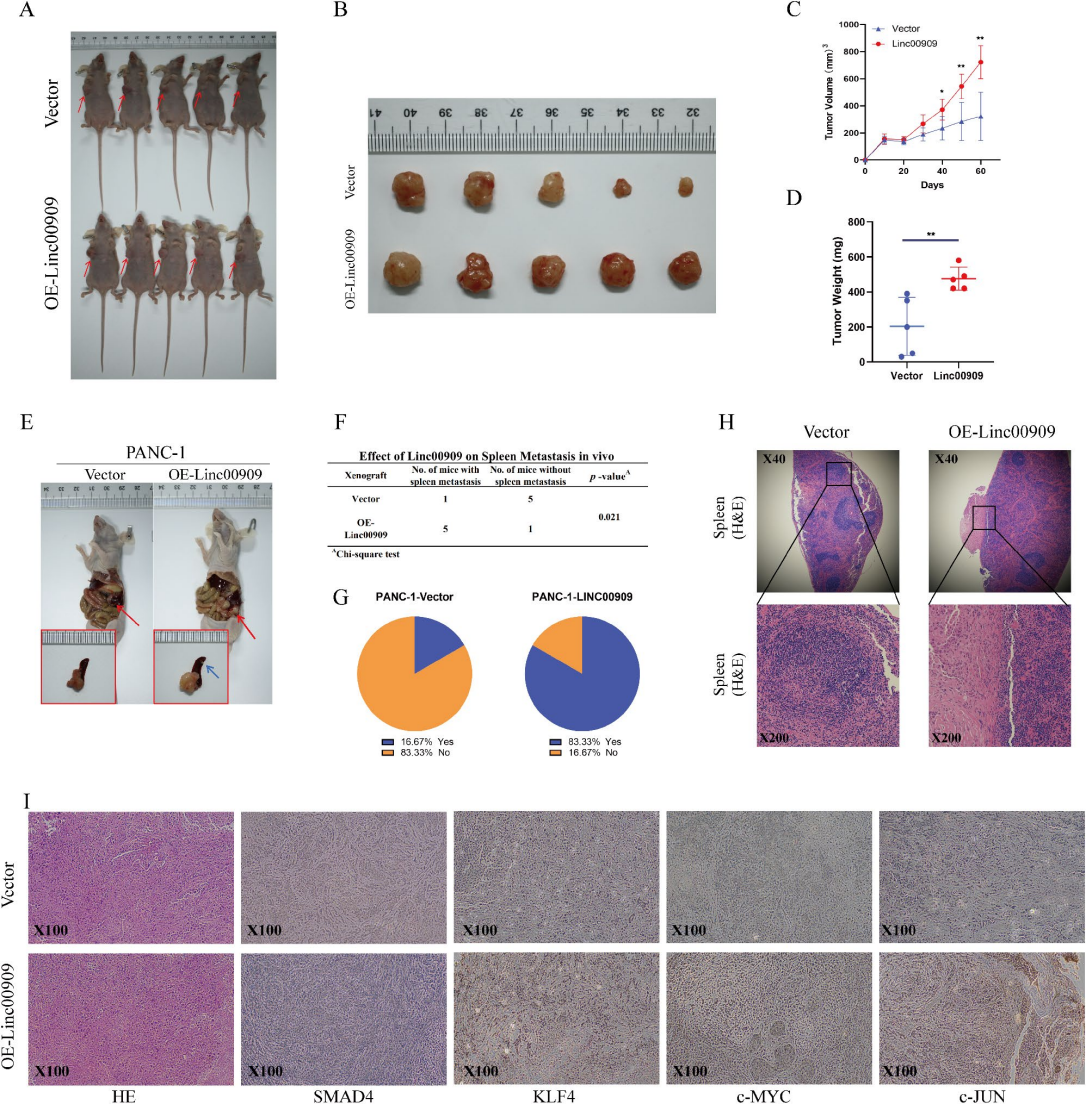
Overexpression of *LINC00909* markedly increases tumor burden and promotes spleen metastasis in pancreatic ductal adenocarcinoma (PDAC). (**A**, **B**) Overall appearance of the subcutaneous xenograft models in the Vector and *LINC00909*-OE groups (*n* = 5 per group). (**C**) Tumor volume curves of the subcutaneous xenografts in the PANC-1/Vector and PANC-1/*LINC00909*-OE groups (*n* = 5). Tumor volume was measured once every 10 days. (**D**) Tumor weights (*n* = 5). (**E**) Representative appearance of orthotopic xenograft models in the Vector and *LINC00909*-OE groups (red arrow: orthotopic pancreatic tumor; blue arrow: metastasis in the spleen). (**F**) The spleen metastasis amount in the orthotopic xenograft model was computed for the indicated group (*n* = 6 per group). (**G**) Pie chart revealing the percentage of mice with spleen metastasis in these two groups. (**H**) **H**&**E** staining of the spleen section. (**I**) IHC staining of *SMAD4*, *KLF4*, *c-Myc*, and *c-JUN* in orthotopic pancreatic tumors obtained from the control and *LINC00909*-OE groups. All *P < 0.05; **P < 0.01. *P*-values were assessed using ANOVA and two-tailed *t*-tests, followed by Dunnett’s tests for multiple comparisons in (**C**, **D**, and **F**)

In order to investigate whether *LINC00909* functions as an oncogene in vivo, PANC-1/*LINC00909*-OE cells and PANC-1/Vector cells were injected subcutaneously into the abdomen of BALB/c-nude mice (number = 5 per group) (Fig. 7A). The results showed an apparent increase in tumor volume as well as tumor weight in the *LINC00909-*OE group compared with the empty control group. The findings affirmed that *LINC00909*-OE enhances tumor growth in PDAC (Fig. 7B–D).

We also aimed to mimic the clinical physiology of PDAC. Thus, we established an orthotopic xenograft model to investigate the effects of *LINC00909* on tumor metastasis. By analyzing the pancreas and spleens 6 weeks after cancer cell injection, we found the incidence of spleen metastasis was 83.33% (5/6) in the *LINC00909*-OE group and was 16.67% (1/6) of mice in the control group, indicating that *LINC00909* accelerated PDAC metastasis in vivo (Fig. 7E-H). The STRING-protein–protein interaction analysis (https://cn.string-db.org/) suggested interactions between *SMAD4*, *KLF4*, *c-Myc*, and *c-JUN* (**Figs. S4A, B**). Subsequently, IHC assays were conducted to detect these protein levels (Fig. 7I). As expected, the levels of *SMAD4* were distinctly decreased, and the factors associated with stemness and metastasis were significantly elevated in tumor tissues obtained from *LINC00909*-OE mice. These results imply that *LINC00909*-OE enhances the pluripotency of stem cell and promotes PDAC tumor metastasis in vivo.

## Discussion

To some extent, developing therapeutic strategies for PDAC depends on the discovery of novel molecules that regulate stemness and progression. Recently, accumulating evidence revealed that lncRNAs play essential roles in regulating stemness properties and cancer metastasis [17]. In this research, we found that *LINC00909* was upregulated in PDAC in both TCGA + Genotype-Tissue Expression Project (TCGA + GTEx) and GDPH cohorts. Higher expression of *LINC00909* was related to poorer clinicopathological features and patient survival, demonstrating it is an unfavorable biomarker in PDAC. Importantly, OE of *LINC00909* induced the expression of pluripotency factors and promoted the CSC phenotype. This discovery supported a novel role of *LINC00909* in regulating pancreatic CSC phenotype.

In the present research, we found that the effect of *LINC00909* on pluripotency factors was dependent on the inhibition of *SMAD4*. *SMAD4*, also termed *DPC4* (deleted in pancreatic cancer) [18], acts as a vital role in embryonic stem cell pluripotency, tumor progression, etc. Of note, loss of SMAD4 is a major contributor to PDAC tumorigenesis. Recently, Hoshino et al. [19] discovered that KD of SMAD4 enhanced the expression of *NANOG*, *SOX2*, and *OCT4* in PC cells, indicating that *SMAD4* affects cancer stemness. Consistently, we showed that OE of *LINC00909* downregulated *SMAD4*, leading to the induction of pluripotency factors. It is well established that loss of *SMAD4* in PDAC may be due to genetic mutation of deletion. However, our results indicated that the decrease or loss of *SMAD4* may be caused by *LINC00909*. These findings revealed that impaired *SMAD4* signaling could be observed in both mutated or wild-type cases, and restoration of *SMAD4* was a critical strategy for eradicating CSCs and inhibiting PDAC progression.

By performing bioinformatics analysis and immunoblotting, we found that *LINC00909* also activated the MAPK/JNK pathway. Similarly, Ozawa et al. [20] claimed that *SMAD4* deletion was associated with activation of the MAPK/JNK pathway and resistance to cetuximab in head and neck squamous cell carcinoma. Moreover, Tan et al. [21] found that loss of *SMAD4* induced the activation of p21 (RAC1)-activated kinase 3 (*PAK3*)-*JNK*-*JUN* by regulating miRNAs in lung cancer, thereby contributing to the development of metastasis. Hence, in addition to affecting CSCs, we hypothesized that *LINC00909* may promote PC metastasis via MAPK/JNK signaling pathway, which is also due to reduction of *SMAD4.* In the future, it will be of interest to investigate whether MAPK/JNK activation is required for maintaining PC stemness.

It is also important to determine the mechanism through which *LINC00909* downregulates *SMAD4* in PC cells. The localization of lncRNAs is closely associated with their biological functions [22]. LncRNAs localized in the nucleus are associated with the regulation of chromatin and transcription [23]. LncRNAs localized in the cytoplasm may affect post-transcriptional regulation, translational regulation, and signal transduction [24]. This study showed that *LINC00909* was located in the cytoplasm and downregulated the mRNA and protein expression of *SMAD4*, which suggests *LINC00909* regulates *SMAD4* at the post-transcriptional level. Specifically, we found that *LINC00909* decreased the stability of *SMAD4* mRNA. It will be of great significance to investigate the regulation of mRNA stability, in particular whether *LINC00909* binds to *SMAD4* mRNA or other RNA-binding proteins. Unfortunately, it was not possible to perform this analysis in the present study due to difficulty in designing an appropriate probe for *LINC00909* with a large base size.

In addition, it is reported that LncRNAs can cross paths with exons within the protein-coding locus site on the opposite strand, antisense LncRNAs possess an intrinsic capability to control their associated sense genes at the transcriptional and/or post-transcriptional tiers [25, 26]. Similarly, Min Zhou et al. also reported that LncRNA *FAM83H-AS1* has the potential to enhance *FAM83H* expression through the stabilization of its mRNA and then enhanced WNT/β-catenin signal pathway [27]. The alias of *LINC00909* is recognized as *ZNF407-AS1*, which represents the antisense strand of *ZNF407*. However, our study did not explore the potential impact of *LINC00909* on the stability of *ZNF407* mRNA and whether it could consequently facilitate the cancer stemness of tumorigenicity of pancreatic ductal adenocarcinoma (PDAC). Consequently, this manuscript exhibits certain limitations, as it omits the discussion of this aspect. Future investigations can be directed towards this facet to further elucidate the prospective functions of this biological molecular marker.

We constructed an orthotopic xenografts model to validate the effect of *LINC00909* on PC metastasis. Furthermore, in vitro experiments suggested that *LINC00909* affected the viability of PC cells under treatment with gemcitabine, indicating that *LINC00909* was associated with the development of chemo-resistance in PC. Future studies utilizing pre-clinical models should be conducted to investigate whether combined treatment with chemotherapeutic agents and inhibition of *LINC00909* will exert a synergistic anti-cancer effect.

**Fig. 8:**
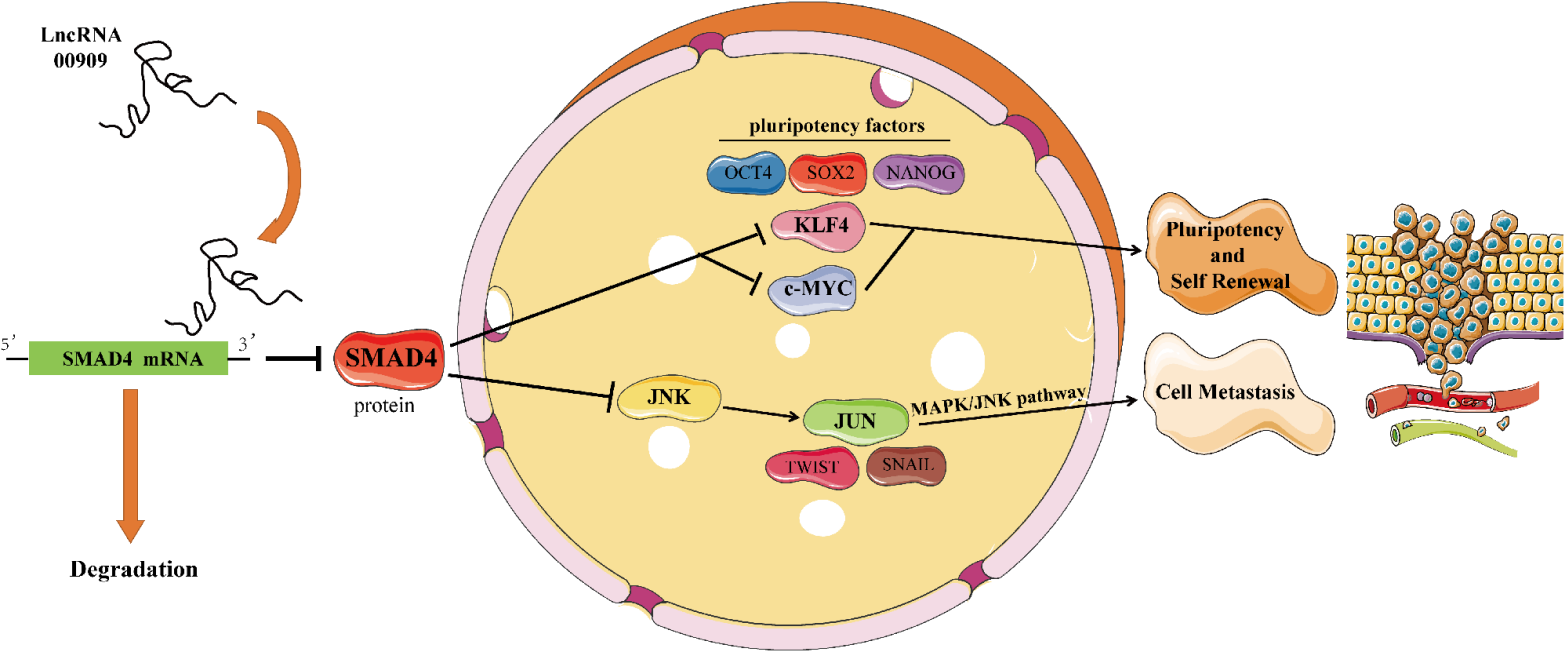
Schematic diagram of the mechanism. By inhibiting *SMAD4* at the post-transcriptional level, *LINC00909* upregulates stemness factors and activates metastasis-related MAPK/JNK signaling pathways

We demonstrated that *LINC00909* is upregulated in PDAC and associated with inferior prognosis. *LINC00909* enhances PDAC cells stemness characteristics and promotes metastasis both in vitro and in vivo. In the mechanism, *LINC00909* inhibits *SMAD4* expression at the post-transcriptional level, thereby upregulating the expression of stemness factors and activating the MAPK/JNK signaling pathway which is associated with metastasis (Fig. 8). Thus, *LINC00909* may be a novel biomarker and target in the treatment of PC.

### Electronic supplementary material

Below is the link to the electronic supplementary material.

**Supplementary Material 1:** Supplementary methods and supplementary figures

## Data Availability

The data in this study are available from the corresponding author upon reasonable request.

## Abbreviations

*LINC00909*: Long intergenic non-protein coding RNA 00909
CSC: cancer stem cell
PC: Pancreatic cancer
PDAC: Pancreatic ductal adenocarcinoma
EMT: Epithelial-to-mesenchymal transition
*KLF4*: Kruppel like factor 4
*OCT4*: Octamer-binding transcription factor 4
*SOX2*: SRY-box transcription factor 2
*NANOG*: Nanog homeobox
ANOVA: analysis of variance
KD: Knockdown
NGS: Next-generation sequencing
OE: Overexpression
DEGs: Differentially expressed genes
GO: Gene Ontology
KEGG: Kyoto Encyclopedia of Genes and Genomes
GSEA: Gene set enrichment analysis
FISH: Fluorescence in situ hybridization
CISH: Chromogenic in situ hybridization
IHC: Immunohistochemistry
FBS: Fetal bovine serum
CCK-8: Cell Counting Kit-8
RT-qPCR: Real-time quantitative Polymerase Chain Reaction
CA19-9: Carbohydrate antigen 19 − 9
GEPIA: Gene Expression Profiling Interactive Analysis
GTEx: Genotype-Tissue Expression Project
GDPH: Guangdong Provincial People’s Hospital
TCGA: The Cancer Genome Atlas
