# Supplementary material for: LINC00909 up-regulates pluripotency factors and promotes cancer stemness and metastasis in pancreatic ductal adenocarcinoma by targeting SMAD4
Source: Biol Direct. 2024 Mar 19;19:24. doi: 10.1186/s13062-024-00463-4 (PMC10949730; doi:10.1186/s13062-024-00463-4)

**Supplementary methods**

Flow cytometry of CSC markers and apoptosis

For the analysis of CSC markers, 1×10^6^ cells were resuspended in staining buffer (BD Biosciences, USA) and blocked with human BD Fc Block (Cat. No. 564219) at 37°C for 20 min. Next, the PDAC cells were incubated with [CD133 (A8N6N) Mouse monoclonal antibody (mAb) (Alexa Fluor® 647 conjugate)](https://www.univ-bio.com/cd133-a8n6n-mouse-mab-flow-specific-alexa-fluor-647-conjugate/4425739.html) and [ALDH1A1 (D9Q8E) XP® Rabbit mAb (phycoerythrin conjugate)](https://www.univ-bio.com/aldh1a1-d9q8e-xp-rabbit-mab-pe-conjugate/397906.html) at 4°C for 30 min. Next, the cells were subjected to flow cytometry for the detection of CSC markers.

For the analysis of apoptosis, after collecting a total of 1×10^6^ PDAC cells, these cells were resuspended in Annexin-V reagent (400 µl). Subsequently, these cells were incubated with Annexin-V phycoerythrin staining solution (5 µl) for 5–10 min and 7-aminoactinomycin D staining solution (5–10 µl) for 1–3 min at 2–8℃. Finally, the cells were analyzed through flow cytometry.

Subcutaneous and orthotopic xenograft tumor models

To evaluate the capacity for tumor growth *in vivo*, PANC-1 cells transfected with empty vector or vector encoding *LINC00909* were subcutaneously injected into the flank abdomen of nude mice (n=5 for each group). We continuously monitored tumor progression for 1 month. Subsequently, all mice were euthanized, and subcutaneous xenograft tumors were removed. Next, tumor formation was observed by measuring tumor weights and volumes.

For the orthotopic xenograft tumor model, the nude mice were anesthetized, and the left abdomen of mice was opened. We injected a total of 1×10^6^ PANC-1 cells (control group or *LINC00909-*OE group) orthotopically into the body of the pancreas and the mice were euthanized 6 weeks after injection. Then, the pancreatic tumors were removed for H&E staining and IHC, and the spleen were removed for H&E staining. All animal experiments were experimented according to the ARRIVE guidelines and authorized by the Institutional Animal Care and Use Committee (IACUC) of GDPH, Guangdong Academy of Medical Sciences.

Statistical analysis

The GraphPad Software (Prism version 9, USA) was used for statistical analyses. Statistical analyses were conducted using the two-tailed Student’s *t*-test or one-way analysis of variance. The data obtained from the wound-healing assays and Transwell assays were analyzed using the ImageJ software (Version 2021, USA). The correlation between *LINC00909* level and clinicopathological features was evaluated using the χ^2^ test or Fisher’s exact test. All statistical analyses were conducted using the GraphPad Software, SPSS version 24.0 (IBM Corp., Armonk, NY, USA) as well as R software version 4.0.1 (https://www.r-project.org/). *P*-values <0.05 demonstrated statistically signiﬁcant differences.

**Supplementary figures**

**Figure S1. Kaplan–Meier analysis of *LINC00909* in several types of cancer.** (A–D) Kaplan–Meier analyses revealed that patients with high *LINC00909* expression and liver hepatocellular carcinoma (LIHC), adrenocortical carcinoma (ACC), brain lower grade glioma (LGG), and bladder urothelial carcinoma (BLCA) had poor overall survival (OS) and disease-free survival (DFS).

*LINC00909*: Long intergenic non-protein coding RNA 00909.


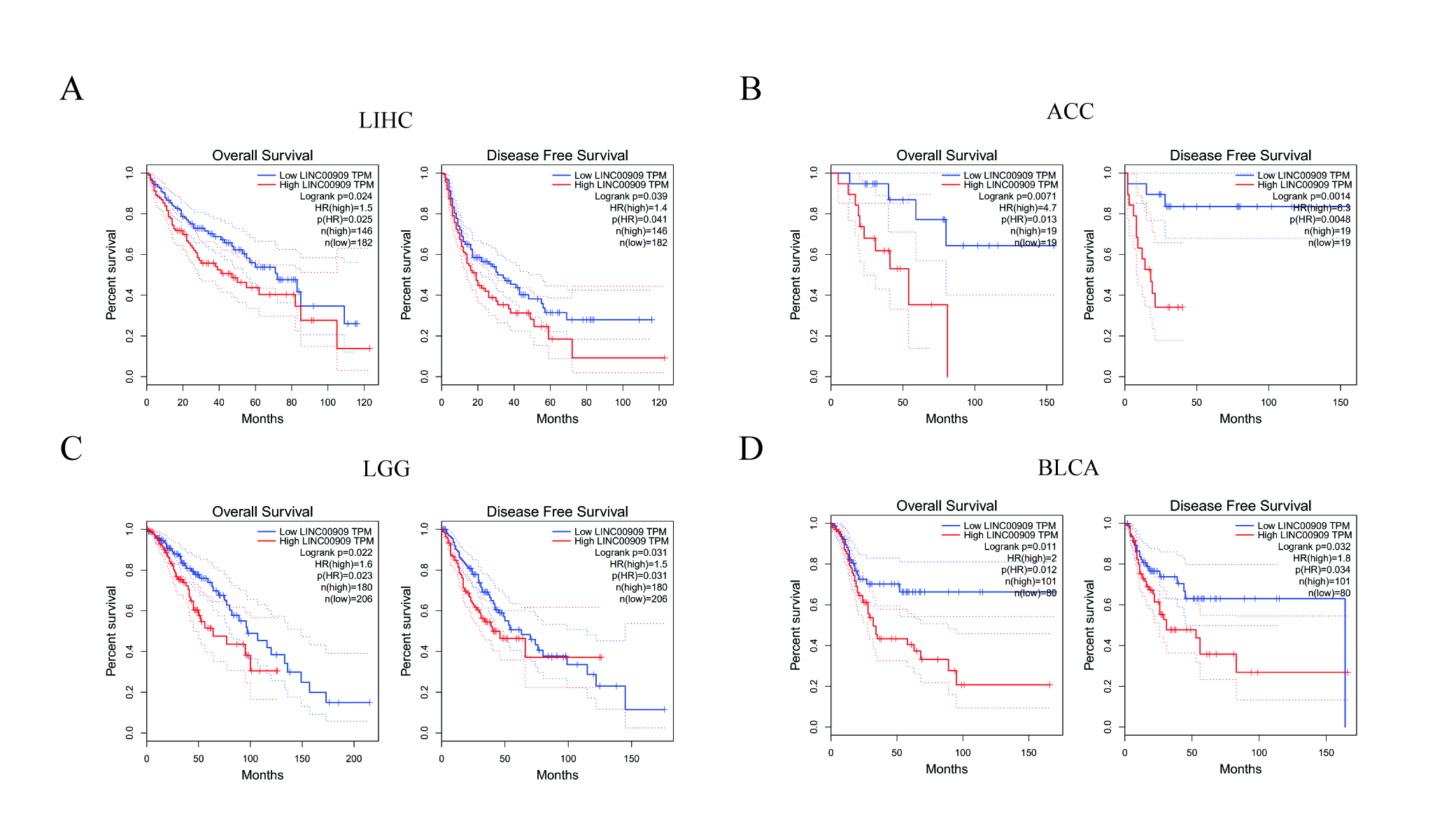


**Figure S2. Wound-healing assays.** (A) Representative images of wound-healing assays after overexpression of *LINC00909* in PANC-1 cells. (B, C) Representative images of wound-healing assays after knockdown of *LINC00909* in PANC-1 (B) and AsPC-1 cells (C). Scale bars: 200 μm. *P*-values were assessed using two-tailed *t*-tests and ANOVA, followed by Dunnett’s tests for multiple comparisons. (****P*<0.001; *****P*<0.0001).

ANOVA: analysis of variance; *LINC00909*: Long intergenic non-protein coding RNA 00909.


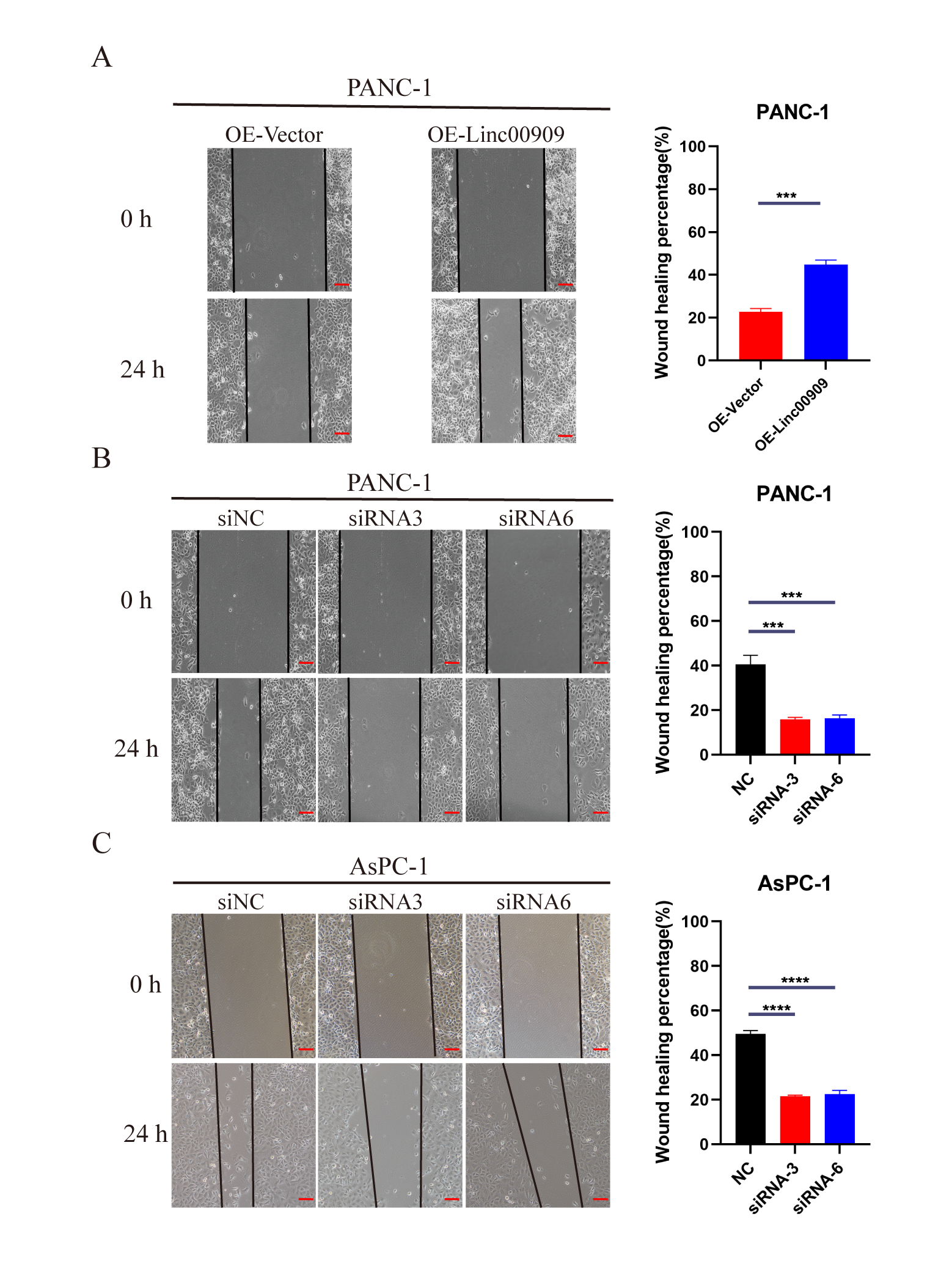


**Figure S3. Correlations of *LINC00909* expression with stemness factors and apoptosis-associated genes in the Gene Expression Profiling Interactive Analysis (GEPIA) Database.** (A) Correlations of *LINC00909* expression with *AKT3*, *BMI1*, and *ALDH1A1* in pancreatic adenocarcinoma (PAAD). (B) Correlations of *LINC00909* expression with *BCL-2*, *BBC3*, *BAK1*, and *BID* in PAAD.

*ALDH1A1*: Aldehyde dehydrogenase 1 family member A1; *AKT3*: AKT serine/threonine kinase 3; *BAK1*: BCL2 antagonist/killer 1; *BBC3*: BCL2 binding component 3; *BCL-*2: B-cell lymphoma-2; *BID*: BH3 interacting domain death agonist; *LINC00909*: Long intergenic non-protein coding RNA 00909.


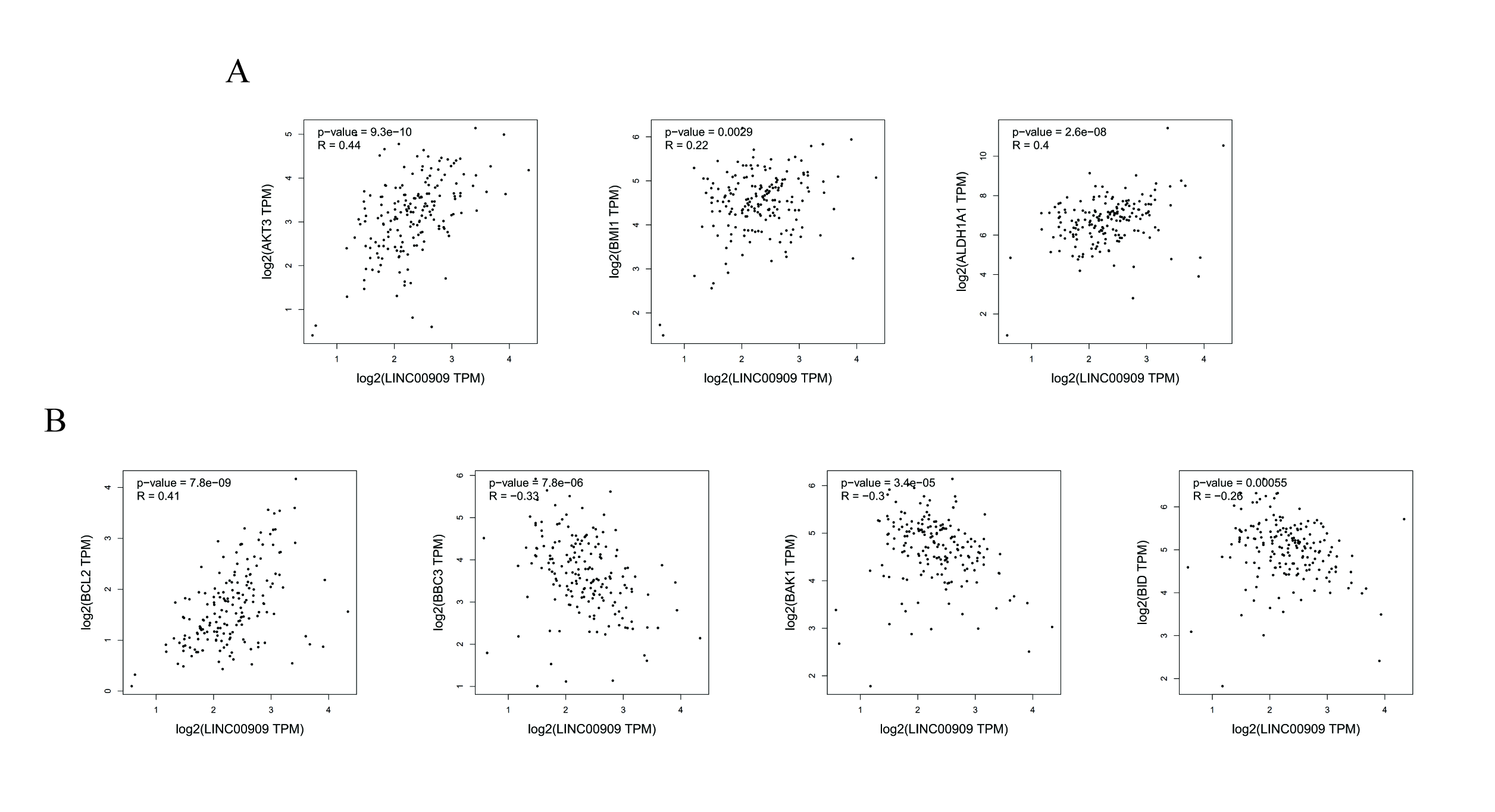


**Figure S4. STRING functional protein association network analysis for *SMAD4* (A) and *KLF4* (B)**.

*KLF4*: Kruppel like factor 4; STRING, Search Tool for the Retrieval of Interacting Genes.


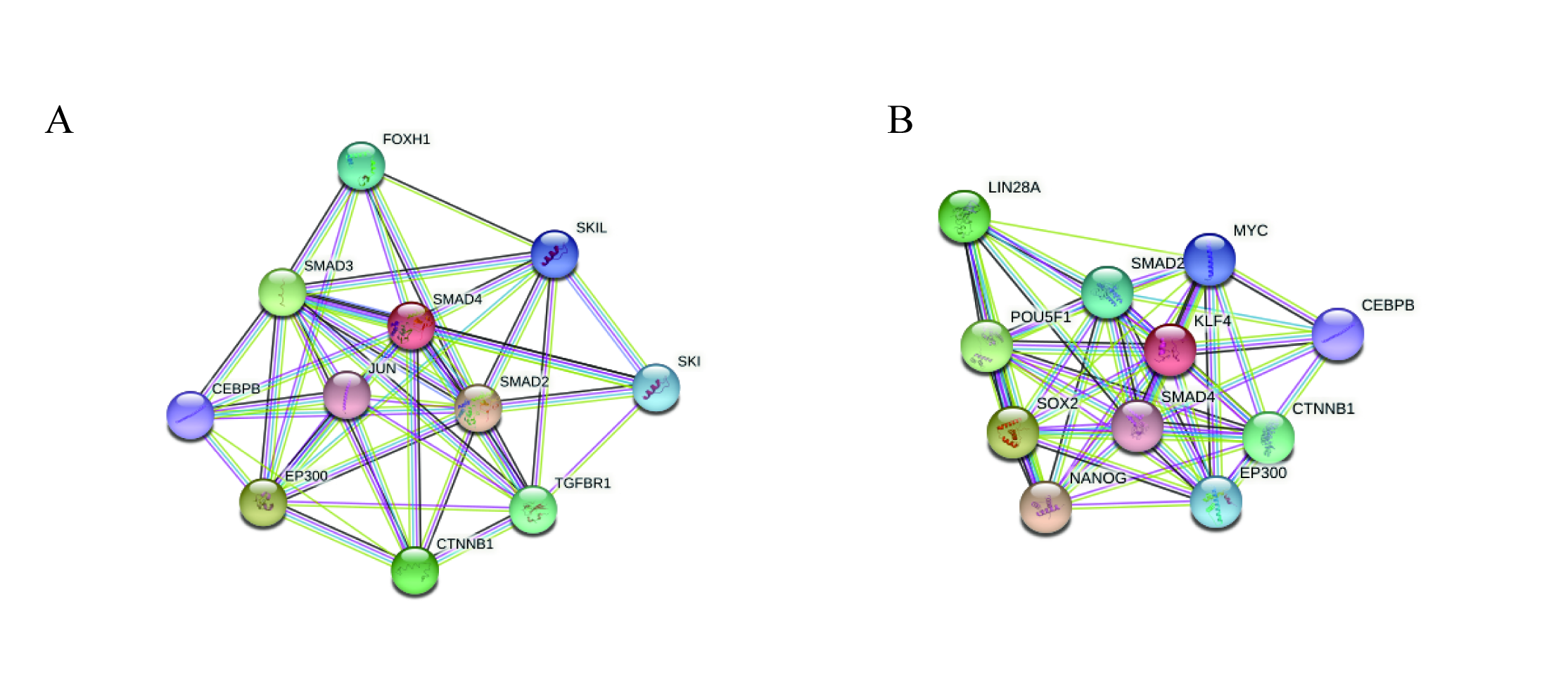

Supplement: Supplementary file 1 — Supplementary Material 1: Supplementary methods and supplementary figures [file 13062_2024_463_MOESM1_ESM.docx]
